# Rapid Implementation of a Diabetes Telemedicine Clinic During the Coronavirus Disease 2019 Outbreak: Our Protocol, Experience, and Satisfaction Reports in Saudi Arabia

**DOI:** 10.1177/1932296820947094

**Published:** 2020-08-07

**Authors:** Mohammed E. Al-Sofiani, Ebtihal Y. Alyusuf, Sahar Alharthi, Abdullah M. Alguwaihes, Reem Al-Khalifah, Assim Alfadda

**Affiliations:** 1 Division of Endocrinology, Department of Internal Medicine, College of Medicine, King Saud University, Riyadh, Saudi Arabia; 2 Division of Endocrinology, Diabetes and Metabolism, The Johns Hopkins University, Baltimore, MD, USA; 3 Strategic Center for Diabetes Research, College of Medicine, King Saud University, Riyadh, Saudi Arabia; 4 Department of Internal Medicine, College of Medicine, King Saud University, Riyadh, Saudi Arabia; 5 Division of Endocrinology, Department of Pediatrics, College of Medicine, King Saud University, Riyadh, Saudi Arabia; 6 Obesity Research Center, College of Medicine, King Saud University, Riyadh, Saudi Arabia

**Keywords:** COVID-19, diabetes, telemedicine, Saudi Arabia

## Abstract

**Background:**

The importance of telemedicine in diabetes care became more evident during the coronavirus disease 2019 (COVID-19) pandemic as many people with diabetes, especially those in areas without well-established telemedicine, lost access to their health care providers (HCPs) during this pandemic.

**Subjects and Methods:**

We describe a simplified protocol of a Diabetes Telemedicine Clinic that utilizes technological tools readily available to most people with diabetes and clinics around the world. We report the satisfaction of 145 patients and 14 HCPs who participated in the virtual clinic and 210 patients who attended the virtual educational sessions about “Diabetes and Ramadan.”

**Results:**

The majority of patients agreed or strongly agreed that the use of telemedicine was essential in maintaining a good glucose control during the pandemic (97%) and they would use the clinic again in the future (86%). A similar high satisfaction was reported by patients who attended the “Diabetes and Ramadan” virtual educational session and 88% of them recommended continuing this activity as a virtual session every year. Majority of the HCPs (93%) thought the clinic protocol was simple and did not require a dedicated orientation session prior to implementing.

**Conclusions:**

The simplicity of our Diabetes Telemedicine Clinic protocol and the high satisfaction reported by patients and HCPs make it a suitable model to be adopted by clinics, especially during pandemics or disasters in resource-limited settings. This clinic model can be quickly implemented and does not require technological tools other than those widely available to most people with diabetes, nowadays. We were able to successfully reduce the number of patients, HCPs, and staff physically present in the clinics during the COVID-19 pandemic without negatively impacting the patients’ nor the HCPs’ satisfaction with the visits.

## Introduction

A global pandemic has been declared by the World Health Organization after cases of coronavirus disease 2019 (COVID-19) were confirmed throughout the world.^1^ To mitigate the spread of the virus, many countries implemented a shelter-in-place order and suspension of operations in nonessential businesses.^2-6^ Routine clinic appointments, including those for patients with diabetes, were cancelled with a short notice, and due to the lack of well-established telemedicine systems in many countries, a large number of patients with diabetes quickly found themselves with little to no medical support during this pandemic.^7-19^

Maintaining an uninterrupted access to health care providers (HCPs) is essential when managing people with diabetes and becomes more important during times of pandemics and disasters.^20-27^ Being confined to home with limited physical activity and hindered access to HCPs and diabetes medications and supply are expected to result in unfavorable metabolic outcomes in people with diabetes.^10,17^ In addition, several reports have linked diabetes to a higher risk of mortality from COVID-19, which added more psychological burden on people with diabetes who are left with no access to their HCPs during a time when this was needed the most.^23,27-29^ Complicating matters, the COVID-19 pandemic broke a few weeks prior to the month of Ramadan, when millions of Muslims, including those with diabetes, attempt to fast every year. Many diabetes clinics in Muslim-majority countries, including Saudi Arabia (SA), arrange a “Pre-Ramadan” clinic visit for their patients during this time to provide diabetes education and medication adjustments prior to fasting.^30^ This could not have been done this year, which further complicated matters for both patients and HCPs.

Though telemedicine is a useful tool to maintain communication between people with diabetes and their HCPs during pandemics, millions of people with diabetes live in developing countries where telemedicine does not exist.^17,31,32^ As the COVID-19 outbreak escalated rapidly, patients and HCPs in many countries were forced to navigate temporary tools to telecommunicate.^11-16^ Despite the lack of telemedicine infrastructure in areas of the world where diabetes is highly prevalent (eg, the Middle East and South Asia), the wide availability of technological resources such as smartphones in these same countries provides an opportunity to quickly adopt a relatively simple telemedicine clinic that could serve the purpose during pandemics without adding a significant burden on patients and health systems. Here, we describe our protocol of a Diabetes Telemedicine Clinic that utilizes tools widely available to patients and HCPs around the world and report the satisfaction of patients and HCPs who experienced this Diabetes Telemedicine Clinic and the virtual “Diabetes and Ramadan” educational sessions.

## Subjects and Methods

The Specialized Diabetes Clinic at King Saud University Medical City serves more than 1000 pediatric and adult patients with diabetes in a traditional care model of “in-person” clinic visits. The clinic is specialized in managing mainly people with type 1 diabetes. In addition, we have one clinic a week focused on managing people with type 2 diabetes who also have cardiovascular disease. As a result, our clinic population is largely young adults with type 1 diabetes. We had no functioning telemedicine clinic prior to the pandemic and our Electronic Medical Record (EMR) system does not have a “Patient Messaging” feature. As the first cases of COVID-19 appeared in SA, we developed a Quality Improvement project, approved by the Institutional Review Board at King Saud University, to implement a Diabetes Telemedicine Clinic. Considering the rapid escalation of the situation, our goal was to design a telemedicine clinic that is simple, practical, and sustainable over a short period of time utilizing tools that were available to us and to our patients at the time.

### Diabetes Telemedicine Clinic Protocol

**Figure 1.**
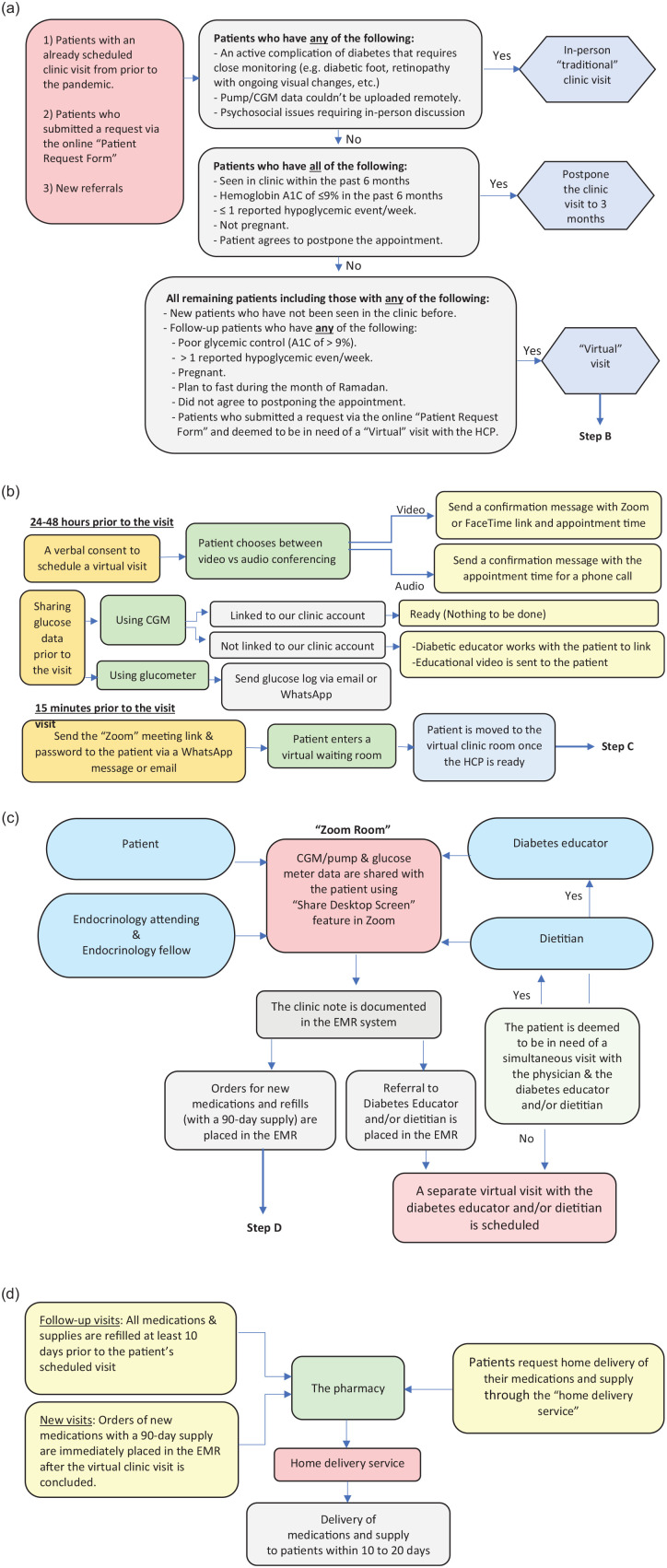
A flowchart highlighting the protocol of the Diabetes Telemedicine Clinic. (a) Patient triage and scheduling the clinic visit. (b) Setting up the virtual clinic visit. (c) Conducting the virtual visit. (d) Ordering and delivering the diabetes medication and supplies.

A flowchart summarizing our Diabetes Telemedicine Clinic Protocol is shown in Figure 1.

A. *Technical Requirements*:

*Hardware*:

A smartphone/computer with audio and video capabilities that can connect to the Internet.

*Software*:

- *A web-based videoconferencing software*: We used Zoom (Zoom, Inc., San Jose, CA, USA) for most visits. Occasionally, FaceTime or audio-only visits were used as per the patients’ requests.
- *Diabetes software*: For patients using continuous glucose monitoring (CGM) systems or insulin pumps, we had already linked their devices and data to our clinic cloud-based accounts as part of the routine clinic practice prior to the pandemic. For those who needed to manually upload the data, we developed an educational video on how to upload data from home and shared the video with all of them.
- *Email/WhatsApp*: Patients using self-monitoring of blood glucose (SMBG) were asked to share a photo of their daily glucose log via email or WhatsApp 24 hours prior to their scheduled visit.
- B. *Online Patient Request Form*:

Since our EMR system does not have a “Patient Messaging” feature, we launched an online “Patient Request” form using the freely available Google Forms. Patients can login to the form, at any time, and submit their requests remotely. Every morning, the patients’ requests get distributed among the HCPs according to the type of request. All requests are addressed within 24 business hours from time of submission.

C. *Scheduling the Visits*:

We contacted all patients who were scheduled to visit our clinic during the pandemic and assigned them to one of three groups: (1) postpone the visit to three months, (2) change the visit to a “virtual” visit, or (3) maintain the in-person “traditional” visit. We used the criteria outlined in Figure 1a to assign the patients to these groups.

D. *Setting up the Virtual Visit*:

*24-48 hours prior to the visit*:

We get a verbal consent from the patients to conduct a virtual visit and send a confirmation message with the appointment time and a link of the “Zoom” application.

*15 minutes prior to the visit*:

A meeting invitation link with a password is sent to patients via a WhatsApp message. The patient is then directed to a “virtual” waiting room until the HCP is ready to begin the visit.

*During the visit*:

To maintain our academic duties, one endocrinology fellow is assigned to each clinic session. The fellow works with the attending endocrinologist in conducting the virtual encounters and documents the notes in the EMR system. To engage the patients in the management discussion and decision-making process, the glucose and insulin pump data are projected on the patients’ computer/phone screen using the “Share Desktop Screen” feature in Zoom.

E.* Shipping the Medications and Supply*:

To minimize patients’ trips to the hospital, our hospital launched a home delivery service to deliver all medications and diabetes supplies to patients. All medication and supply refills are submitted at least 10 days prior to the patient’s scheduled visit to minimize the risk of having patients run out of medications or supplies.

F.* Virtual “Diabetes and Ramadan” Educational Sessions*:

Patients were invited to attend an interactive virtual session about “Diabetes and Ramadan.” We conducted 10 sessions over two weeks that were jointly presented by physicians, diabetes educators, and dietitians, followed by a period of questions and answers, and were attended by 30-90 participants/session. Various topics were discussed in these sessions including risk quantification, glucose monitoring, medication adjustments, fluids and dietary advice, when to break the fast, estimating carb content of meals consumed in Ramadan, and exercise advice. A separate session was given to patients using insulin pump to incorporate specific pump setting adjustment and features that can be utilized in Ramadan.

G. *Patients’ and HCPs’ Satisfaction Surveys*:

Patients completed an anonymous online satisfaction survey after each visit, whereas HCPs completed an anonymous satisfaction survey at the end of the first month of using telemedicine. The patient satisfaction survey was sent out to the first 150 patients who visited the Diabetes Telemedicine Clinic. Patients who attended the “Diabetes and Ramadan” educational sessions completed a separate anonymous satisfaction survey. Respondents rated their level of agreement/disagreement on a five-point Likert scale, and all surveys were completed between March 24 and April 24, 2020.

## Results

### Patient Characteristics

During the first four weeks of establishing the Diabetes Telemedicine Clinic, we conducted over 300 virtual visits and 10 virtual interactive educational sessions on “Diabetes and Ramadan” that was attended by more than 300 patients with diabetes. Of those, 145 of the first 150 patients seen virtually evaluated their experience with the Diabetes Telemedicine Clinic and 210 participants reported their satisfaction with the “Diabetes and Ramadan” virtual educational session. The baseline characteristics of the patients who attended our Diabetes Telemedicine Clinic and evaluated their experience are presented in Table 1.

**Table 1.** Characteristics of Patients Who Visited the Diabetes Telemedicine Clinic and Completed the Satisfaction Form.

	All (*n* = 145)	Video conferencing (*n* = 100)	Audio conferencing (*n* = 45)	*P*-value (video vs audio conferencing)*
Age, median (IQR), years	21 (11)	20 (8.5)	24 (15)	.12
Female, *n* (%)	98 (68)	72 (72)	26 (58)	.09
Types of diabetes, *n* (%)				
T1D	129 (88.97)	91 (91)	38 (85)	.23
T2D	15 (10.34)	9 (9)	6 (13)	
Diabetes in pregnancy	1 (0.69)	0 (0)	1 (2)	
Most recent A1c, median (IQR), %	8.9 (3.8)	9.4 (3.85)	8.4 (2.40)	.15
Visiting from out of Riyadh, *n* (%)	24 (17)	17 (17)	7 (16)	.83
Use of diabetes technology, *n* (%)				
Using CGM, *n* (%)	112 (77.24)	78 (78)	34 (75.56)	.75
Using insulin pump, *n* (%)	32 (22.07)	21 (21)	11 (24.44)	.64
Type of visit				
New patient, *n* (%)	17 (12)	14 (14)	3 (7)	.20
Follow-up visit, *n* (%)	128 (88)	86 (86)	42 (93)	
First-time user of telemedicine, *n* (%)	116 (80)	76 (76)	40 (89)	.07
HCP conducting the visit				
With endocrinologist, *n* (%)	92 (64)	58 (59)	34 (76)	.03^†^
With diabetes educator, *n* (%)	24 (17)	22 (22)	2 (4)	
With dietitian, *n* (%)	28 (19)	19 (19)	9 (20)	

Abbreviations: IQR, interquartile range; CGM, continuous glucose monitor; HCP, health care provider; T1D, type 1 diabetes; T2D, type 2 diabetes.

* Differences between video and audio conferencing were examined using *t* tests for normally distributed continuous data and Kruskal–Wallis testing for non-normally distributed continuous data; categorical variables were examined using chi-squared tests of homogeneity.

† Statistically significant difference (*P*-value <.05) between video and audio conferencing.

### Online Requests Submitted by Patients During the Pandemic

**Figure 2.**
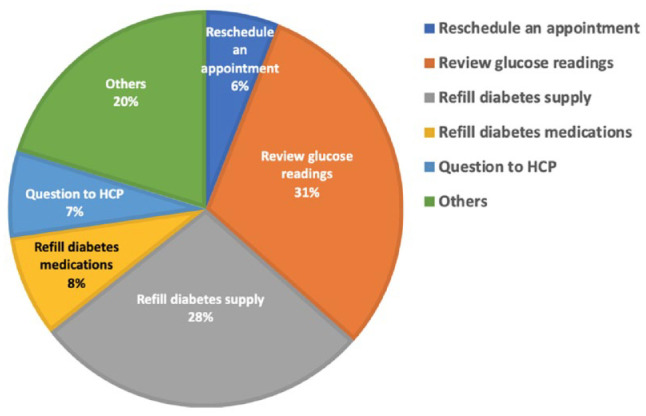
The proportion of online requests submitted by the patients from March 24 to April 24, 2020. Most frequent online request submitted by patients was “review glucose readings and adjust medications” (31%), followed by “refilling the diabetes supply” (28%); “others” which included questions about carbohydrate counting, device malfunction, and questions about diet (20%); “refilling diabetes medications” (8%); “question to the HCP” (7%); and “reschedule an appointment” (6%). Abbreviation: HCP, health care provider.

We have received over 450 patients’ requests through the online “Patient Request” during the first month of establishing the Diabetes Telemedicine Clinic. The most frequently submitted request by patients was to review the glucose readings and adjust medications (31%). The distribution of the other requests is shown in Figure 2.

### Patients’ and HCPs’ Satisfaction with the Diabetes Telemedicine Clinic

**Figure 3.**
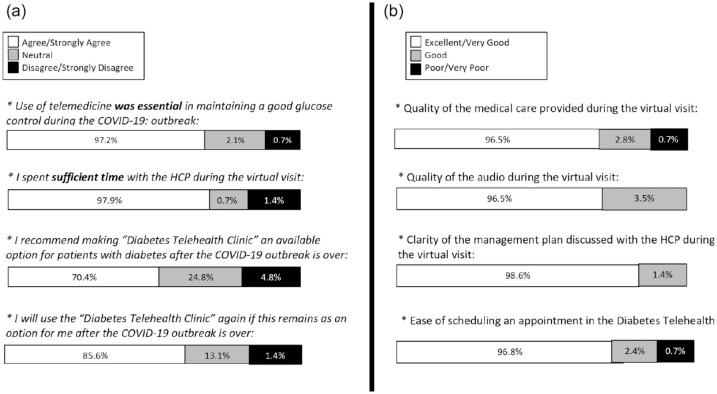
Patients’ satisfaction with the Diabetes Telemedicine Clinic. (a) Level of patients’ agreements with the following statements: “Use of telemedicine was essential in maintaining a good glucose control during the COVID-19: outbreak,” “I spent sufficient time with the HCP during the virtual visit,” “I recommend making Diabetes Telemedicine Clinic an available option for patients with diabetes after the COVID-19 outbreak is over,” and “I will use the Diabetes Telemedicine Clinic again if this remains as an option for me after the COVID-19 outbreak is over.” (b) Patients rating of the quality of the following services: “Quality of the medical care provided during the virtual visit,” “Quality of the audio during the virtual visit,” “Clarity of the management plan discussed with the HCP during the virtual visit,” and “Ease of scheduling an appointment in the Diabetes Telemedicine.” Abbreviations: COVID-19, coronavirus disease 2019; HCP, health care provider.

The majority of our patients agreed or strongly agreed that the use of telemedicine is essential in achieving good glucose control during the COVID-19 outbreak (97%); the HCP spent sufficient time with them during the virtual visit (98%), and they will use the “Diabetes Telemedicine Clinic” again in the future even after the COVID-19 outbreak is over (86%). The rest of the results is shown in Figure 3. The majority of HCPs agreed or strongly agreed that the Diabetes Telemedicine Clinic protocol was simple enough and does not require technical skills or dedicated orientation session prior to working there (93%); the clinic almost always met its patients care treatment goals (71%), and the time spent with patients during the virtual visit was sufficient (93%). The remaining results of the survey are shown in Table 2. The advantages of the Diabetes Telemedicine Clinic most frequently reported by our patients are as follows: telemedicine minimized my risk of acquiring infection (74.4%), less waiting time to see the HCP (72.4%), no need to make a trip to the clinic (64.8%), same quality of care received as in the traditional clinic (64.8%), and cost saving (16.5%). Very few patients reported disadvantages of the Diabetes Telemedicine Clinic as follows: telemedicine requires technical skills (13.1%), care quality in the telemedicine clinic is less than that in the traditional clinic (6.9%), risk of loss of privacy (2.7%), and telemedicine is more expensive than in-person clinic (0.7%) (data not shown).

**Table 2.** Health Care Providers’ Satisfaction with the Diabetes Telemedicine Clinic (*n* = 14).

Statement	Strongly agree/agree	Neutral	Disagree/strongly disagree
It was easy to run and work in the Diabetes Telemedicine Clinic.	10 (71.4%)	2 (14.3%)	2 (14.3%)
I was confident and felt at ease when I worked in the Diabetes Telemedicine Clinic.	11 (78.6%)	1 (7.1%)	2 (14.3%)
The images and audios during the Telemedicine encounter were clear.	11 (78.6%)	2 (14.3%)	1 (7.1%)
The Diabetes Telemedicine Clinic almost always met its patient care treatment goals.	10 (71.4%)	3 (21.4%)	1 (7.1%)
I believe the Diabetes Telemedicine Clinic was essential in maintaining a good glucose control for our patients during the COVID-19 pandemic.	13 (92.9%)	1 (7.1%)	0
The quality of care provided in the Diabetes Telemedicine Clinic was excellent.	11 (78.6%)	2 (14.3%)	1 (7.1%)
The time spent with patients when working in the Diabetes Telemedicine Clinic was sufficient.	13 (92.9%)	0	1 (7.1%)
In the Diabetes Telemedicine Clinic, the number of patients that I can see virtually in one clinic is more than the number that I can see in the standard “in-person” clinic.	4 (28.6%)	5 (35.7%)	5 (35.7%)
In the Diabetes Telemedicine Clinic, there is less No-Shows rates among the patients with CONFIRMED appointments compared to that in the standard in-person clinic.	5 (35.7%)	8 (57.1%)	1 (7.1%)
The Diabetes Telemedicine Clinic protocol is simple enough that it does not require technical knowledge or skills, and HCPs do not need to attend a dedicated orientation session prior to working there.	13 (92.8%)	1 (7.1%)	0
My experience in the Diabetes Telemedicine Clinic would have been more satisfying if I had an orientation session on how to conduct a Telemedicine visit.	4 (28.6%)	5 (35.7%)	5 (35.7%)
For the most, I am satisfied with my experience with the Diabetes Telemedicine Clinic.	10 (71.4%)	3 (21.4%)	1 (7.1%)
Some of our patients will still benefit from offering the Diabetes Telemedicine Clinic after the COVID-19 pandemic is over.	14 (100%)	0	0
I will use the Diabetes Telemedicine Clinic for some of my patients in the future if this is made as an available option in our clinics.	13 (92.8%)	1 (7.1%)	0

Abbreviations: COVID-19, coronavirus disease 2019; HCP, health care provider.

### Patient Satisfaction with the “Diabetes and Ramadan” Virtual Sessions

Of the 210 participants who evaluated the “Diabetes and Ramadan” interactive virtual educational sessions, 99% of the respondents thought that the relevance and quality of the information discussed during the session were excellent or very good and 97% thought that the amount of knowledge gained from attending the session was excellent or very good. Interestingly, 88% of the respondents agreed or strongly agreed that this session should be continued as a virtual visit every year, even after the COVID-19 situation is over. The remaining results of the survey are shown in Table 3.

**Table 3.** Patients Satisfaction with the Interactive Live Online Session on “Diabetes and Ramadan” (*n* = 210).

	Agree/strongly agree	Neutral	Disagree/strongly disagree
The “Diabetes and Ramadan” online session is essential in helping people with diabetes maintain a good glucose control during Ramadan:	97.1%	2.9%	0%
I recommend continuing the “Diabetes and Ramadan” session as an online meeting every year, after the COVID-19 outbreak is over:	88.1%	9%	2.9%
	Excellent/very good	Good	Poor/very poor
The amount of knowledge I gained from the “Diabetes and Ramadan” online session was:	97.1	1.9%	1%
The relevance and quality of information discussed during the “Diabetes and Ramadan” online session was:	99%	1%	0%
The video and audio quality of the “Diabetes and Ramadan” online session was:	96.6%	2.9%	0.5%

## Discussion

Our clinic was one of many clinics around the world that had no telemedicine infrastructure or prior experience with this model of care and found themselves navigating the process of transitioning to become fully virtual as the COVID-19 situation evolved. Though most of our patients have not used telemedicine prior to this time, the extremely high use of smart devices and wide availability of access to the internet, including in remote areas in SA, made our transition to telemedicine a relatively smooth one.^33^ Moreover, diabetes care is an area that is well suited to the use of telemedicine,^31^ especially with the evolving advances in glucose monitoring devices and remote glucose data sharing features. Even for patients who still depend on SMBG, they can use Bluetooth glucose meters that allow for data upload to the cloud, or they can simply send photos of their daily glucose log to the HCPs via email or phone texts.

The high number of online “Patient Requests” submitted during the first month of the pandemic shows that people with diabetes are in fact ready to utilize telemedicine when this is made available. Third of the requests were asking for an HCP to review their glucose readings and make treatment adjustments, which also shows that people with diabetes crave for an uninterrupted communication with their HCPs and highlights their interest in utilizing telemedicine to improve their glucose control. Moreover, the remarkably high patients’ attendance at the Diabetes Telemedicine Clinic and the virtual “Diabetes and Ramadan” educational sessions along with the high satisfaction reported with the overall quality of the virtual visits and desire to continue this care model in the future highlight the efficiency of our protocol despite its simplicity. Prior studies have shown similar high patients’ satisfaction with telemedicine,^34^ and clinical outcomes of patients using telemedicine services were found to be comparable with those using traditional “in-person” clinic visits.^35^ However, these studies were done in the United States, where telemedicine is better established than it is in SA and many other countries around the world. Nonetheless, our results show that a simplified protocol of a Diabetes Telemedicine Clinic can serve the purpose and result in a similarly high patients’ satisfaction when implemented in countries that lack the infrastructure of telemedicine.

In our experience, patients felt more valued and empowered when HCPs reached out to them and offered to deliver the diabetes care remotely. The patients and HCPs had a general feeling that the visits have become more patient centered in the Diabetes Telemedicine Clinic compared to how it was in the traditional “in-person” clinic. Patients played an active role during the virtual visits, as they had to send their daily glucose log to their HCPs prior to the visit, learn how to upload the data from their devices, and submit online requests and questions to the clinic. It was also noted that many virtual visits in the pediatric clinic were attended by both parents and other key family members, which increases the family engagement in the child care compared to the standard “in-person” visits. All of these factors have likely played a role in the high satisfaction reported by our patients.

There are many points that we learned throughout this process. It is essential that HCPs always remember to introduce themselves, by stating their names and roles, and confirm the patient identity prior to starting the virtual encounter. As we aim to improve the quality of care provided to patients using telemedicine, it is essential to maintain high standards of patients’ safety and take all necessary precautions to protect against the loss of patient’s privacy. We have also learned that it is important to educate people with diabetes who use insulin pumps or CGMs on how to upload their data from home. This skill not only empowers patients but also becomes valuable during pandemics and other situations when patients are not able to make the trip to the clinic. Patients who might be anxious about using technology (eg, older adults or those who are hard-of-hearing) may find telemedicine difficult to adopt; however, having a relative or a friend accompanying them during the virtual visit makes it smoother and results in a high patient’s satisfaction based on our experience. As part of the verbal consent to participate in a telemedicine clinic, patients should be made aware of the limitations of virtual visits, including the inability to perform a complete physical examination, beyond the inspection for acanthosis nigricans, lipohypertrophy, and other visible signs. Physical examination is an important component of diabetes care, particularly for new patients who are seen by HCPs for the first time. Similarly, patients who require laboratory testing that cannot be delayed (eg, basic metabolic panel before and after initiating angiotensin-converting enzyme inhibitors or angiotensin receptor blockers) or routine laboratory testing (eg, hemoglobin A1C or lipid panel) will still have to go to a laboratory center to get their blood samples drawn. Therefore, we view the future of telemedicine in diabetes care as being a valuable complementary tool to the traditional “in-person” visits, rather than a replacement.

## Conclusion

We outlined the details of our protocol of a Diabetes Telemedicine Clinic for resource-limited settings along with practical tips on how this virtual clinic can be rapidly implemented utilizing tools that are available, nowadays, for most patients and HCPs around the world. We showed the high satisfaction of our patients and HCPs with the Diabetes Telemedicine Clinic and their desire to maintain this care model after the COVID-19 situation is over. As a result of adopting the Diabetes Telemedicine Clinic, we were able to successfully reduce the number of patients, HCPs, and staff physically present in the clinics without negatively impacting the quality of care provided to our patients nor their satisfaction with the visits. Though we hope that our quick adoption of a Diabetes Telemedicine Clinic during the COVID-19 pandemic will translate into a clinically meaningful impact on patients who attended the clinic, this will need to be examined in future studies.
